# Expression of Progesterone Receptor Membrane Component 1 (PGRMC1), Progestin and AdipoQ Receptor 7 (PAQPR7), and Plasminogen Activator Inhibitor 1 RNA-Binding Protein (PAIRBP1) in Glioma Spheroids* In Vitro*

**DOI:** 10.1155/2016/8065830

**Published:** 2016-06-01

**Authors:** Juraj Hlavaty, Reinhard Ertl, Ingrid Miller, Cordula Gabriel

**Affiliations:** ^1^Institute of Anatomy, Histology and Embryology, University of Veterinary Medicine, 1210 Vienna, Austria; ^2^VetCORE, Facility for Research, University of Veterinary Medicine, 1210 Vienna, Austria; ^3^Institute of Medical Biochemistry, University of Veterinary Medicine, 1210 Vienna, Austria

## Abstract

*Objective.* Some effects of progesterone on glioma cells can be explained through the slow, genomic mediated response* via* nuclear receptors; the other effects suggest potential role of a fast, nongenomic action mediated by membrane-associated progesterone receptors.* Methods.* The effects of progesterone treatment on the expression levels of progesterone receptor membrane component 1 (PGRMC1), plasminogen activator inhibitor 1 RNA-binding protein (PAIRBP1), and progestin and adipoQ receptor 7 (PAQR7) on both mRNA and protein levels were investigated in spheroids derived from human glioma cell lines U-87 MG and LN-229.* Results.* The only significant alteration at the transcript level was the decrease in PGRMC1 mRNA observed in LN-229 spheroids treated with 30 ng/mL of progesterone. No visible alterations at the protein levels were observed using immunohistochemical analysis. Stimulation of U-87 MG spheroids resulted in an increase of PGRMC1 but a decrease of PAIRBP1 protein. Double immunofluorescent detection of PGRMC1 and PAIRBP1 identified the two proteins to be partially colocalized in the cells. Western blot analysis revealed the expected bands for PGRMC1 and PAIRBP1, whereas two bands were detected for PAQR7.* Conclusion.* The progesterone action is supposed to be mediated* via* membrane-associated progesterone receptors as the nuclear progesterone receptor was absent in tested spheroids.

## 1. Background

Glioblastoma multiforme (GBM, grade IV astrocytoma) is the most common and most aggressive malignant primary brain tumor in adults [1]. An effective treatment for GBM is not existent; the standard therapy is a combination of surgical resection of the tumor and subsequent chemotherapy with severe side effects resulting in a maximal increase of survival time for two months [2–4]. Therefore, improvement of the knowledge concerning this type of brain tumor to identify targets and therapeutic agents is voraciously needed.

Based on the knowledge that men are more often affected by primary GBM than women only until the age of menopause [5–7], a potential function of sex steroid hormones in GBM development was investigated in different studies.

In 2015, Atif et al. identified the steroid hormone progesterone as potential promising therapeutic agent in GBM [8]. In their study, the dose-dependent antitumor effects of progesterone were tested in well-established glioma cell lines* in vitro* and in subcutaneous U-87 MG xenografts in murine models* in vivo* [8]. Progesterone was already known to have beneficial effects on the outcome of brain injuries accompanied with cerebral edema and inflammation [9] and known to feature dose-dependent antiproliferative and proapoptotic effects in other tumors including breast, ovarian, and endometrial cancer [10, 11]. Although these effects were observed and documented, the background of progesterone mediated response in tumor cells is not fully elucidated. The action of progesterone depends on different mechanisms including a slow, genomic mediated response* via* nuclear progesterone receptors (nPGR) and a fast, nongenomic action, which can be mediated* via* membrane-associated progesterone receptors (MAPRs) [12–14]. Some effects of progesterone in glioma cells can be mediated* via* the nuclear receptors but other cannot, suggesting a potential role of the MAPRs. Members of the MAPRs were localized in different regions of the rat brain [15]. Furthermore, it was demonstrated that the sex steroid hormones 17*β*-estradiol and progesterone influence the expression of MAPRs in the brain [16]. In neuroendocrinology, it was shown that in the hippocampus the classical nPGR responded differently to estrogen and progesterone supplementation compared to PGRMC1 during the rat estrous cycle [17]. Furthermore, PGRMC1 knockdown in microglia suppressed the progesterone 17*β*-estradiol antagonism of neurite outgrowth in female rat brain [18]. In different cells and tissues, it has been reported that PGRMC1 is associated with cell cycle regulation including proproliferative and antiapoptotic effects in response to progesterone [19–22]. Therefore, the close interaction of progesterone mediated effects and PGRMC1 expression, especially in glia cells, is supposed to be a potential target to study the nPGR-independent effects of progesterone on glioma cells and, thereby, PGRMC1 was selected as protein of interest in the present study.

Mediating the responsiveness to progesterone via PGRMC1 was assumed to be partially depending on plasminogen activator inhibitor 1 RNA-binding protein (PAIRBP1), also known as SERPINE1 mRNA-binding protein (SERBP1) [19, 23], which was also identified to be expressed in the different regions of the rat brain [15]. PAIRBP1 participates in the antiapoptotic actions of progesterone in spontaneously immortalized granulosa cells, but a recent study identified progesterone binding being independent from its interaction with PAIRBP1 [19]. Based on these results, it is suggested that PAIRBP1 is not a component of the membrane progesterone receptor but instead is an involved downstream component of the signaling pathway leading to protection against apoptosis and was therefore selected to be a further protein of interest in the present study.

Additionally, the MAPR family member PAQR7 (mPR*α*) has to be investigated in detail as a potential binding partner for PGRMC1 because comparable antiapoptotic progestin actions mediated through these two membrane proteins were identified [24, 25]. Recent studies demonstrated that progesterone-induced alterations of different genes involved in the antiapoptotic pathways include both PAQR7 and PGRMC1 [22, 26] and that PGRMC1 and PAQR7 together with PGRMC2 form a complex within the cytoplasm which seems to be required for P4's action [27]. Progesterone dependent PI3K/Akt/mTOR signaling modulation was observed in glioma cells* in vitro* although the nPGR was blocked by RU486, an inhibitor of the nPGR, suggesting that the nongenomic action of progesterone via MAPRs has an important role in the progesterone responsiveness of glioma cells [8].

Therefore, the aim of the study was to investigate the effects of different concentrations of progesterone on PGRMC1, PAIRBP1, and PAQR7 expression in glioma cell spheroids on mRNA and protein levels. Two different cell lines were used to identify potential differences between GBM cells of female (LN-229) and male (U-87 MG) origin. The application of a three-dimensional glioma cell spheroid model was relevant to mimic the natural tumor situation in more detail compared to a monolayer cell culture [28].

## 2. Materials and Methods

### 2.1. Cell Lines and Cell Culture

The human glioma cell lines LN-229 and U-87 MG were obtained from LGC Promochem (CRL-2611) and Cell Line Service (CLS# 300367), respectively. The short tandem repeat (STR) analysis performed at the Cell Culture Facility of the Medical University of Graz, Austria, confirmed the cell lines' identity. The cells were maintained in Dulbecco's modified Eagle's medium containing 4.5 g/L glucose (DMEM, Sigma) supplemented with 10% heat-inactivated fetal calf serum (FCS, Sigma) and antibiotic-antimycotic mix (BioSell), hereafter referred to as growth medium, in a humidified atmosphere with 5% CO_2_ at 37°C.

Spheroids were grown by inoculating 2 × 10^6^ cells in nonadherent 90 mm petri-dishes (Rotilabo® Petrischalen, ROTH) in 10 mL of growth medium in the absence of additional scaffolds and matrices. Clusters of cells were observed 24 hours after seeding. The medium was changed every second day until spheroids reached day 14. Progesterone (Sigma) stimulation at the concentration previously used by Ramaswamy (3 ng/mL or 30 ng/mL equal to 9.54 nM or 95.4 nM of progesterone) was performed for the last two days of cultivation (D13, D14) with medium exchange every day [29]. Four independent biological replicates per group were prepared and used for further analysis unless otherwise indicated.

Spheroids were harvested by centrifugation at room temperature (1000 rpm for 3 min), washed with PBS (Sigma), and either prepared for histological analysis as described below or stored as dry pellet at −80°C until being further processed.

### 2.2. RT-qPCR

Spheroid pellets were resuspended in 600 *μ*L QIAzol lysis reagent (Qiagen, Hilden, Germany) and mechanically homogenized with MagNA Lyser instrument (Roche, Rotkreuz, Switzerland) using 1.2 mm ceramic beads at 6000 rpm for 20 sec. Subsequently, the homogenized samples were chilled on ice and centrifuged (12 000 ×g for 1 min) and the cellular RNA was extracted using the Direct-zol RNA Miniprep Kit (Zymo Research, Irvine, USA) following the recommended protocol. All RNA samples were treated with DNase I (Turbo DNA-Free Kit, Ambion, Austin, USA) to remove contaminating DNA. RNA quality control was performed on the Agilent 2100 Bioanalyzer using the RNA Nano 6000 Kit (Agilent Technologies, Santa Clara, USA). The measured RNA integrity numbers ranged from 8.3 to 10. The RT-qPCR primers for the target gene PAQR7 and the reference gene RPL27 were taken from the literature [30, 31]. Hydrolysis probes were designed for the existing primer pairs to increase the target specificity (Supplementary Table 1 in Supplementary Material available online at http://dx.doi.org/10.1155/2016/8065830). Additional RT-qPCR assays were designed for PGRMC1 and PAIRB1 using the PrimerExpress 2.0 software (Life Technologies, Carlsbad, USA). All assays were validated by the generation of standard curves and the calculation of PCR reaction efficiencies using the formula described in Klein [32]. For RT-qPCR, 1 *μ*g total RNA was retrotranscribed into cDNA utilizing the High Capacity Reverse Transcription Kit (Life Technologies) according to the manufacturer's instructions. Minus reverse transcription controls (samples in which no RT enzyme was added) were included for all RNAs to monitor the amplification of residual DNA. RT-qPCR reactions were done in 20 *μ*L mixes including 12.5 ng cDNA template, 0.2 mM of each dNTP, 3 mM MgCl_2_, 1x buffer B2 (Solis BioDyne, Tartu, Estonia), 300 nM of each primer, 200 nM probe, 50 nM ROX reference dye (Biotium, Hayward, USA), and 1-unit HOT FIREPol DNA polymerase (Solis BioDyne). All samples were analyzed in duplicates on a Viia7 Real-Time PCR System (Life Technologies) using the following temperature profile: initial denaturation at 95°C for 10 min, 45 cycles of 95°C for 15 sec, and 60°C for 1 min. The expression stability of RPL27 was assessed with the BestKeeper analysis tool [33]. Target gene expression levels were then normalized to those of the reference gene (RPL27) and relative expression changes were calculated using the comparative 2^−ΔΔCT^ method [34].

### 2.3. RT-PCR

The expression of the nuclear progesterone receptor (nPGR) was investigated by reverse transcription PCR (RT-PCR). Two previously described RT-PCR assays were used to ensure the detection of all known PGR transcript isoforms A–D (accession numbers NM_000926.4, NM_001202474.3, NM_001271161.2, and NM_001271162.1). The first assay named “PGR,” by Latil [35], detects the isoforms A–C, whereas the second RT-PCR “PR-A + B” [36] amplifies A, B, and D (Supplemental Table 1). The reaction mixes for RT-PCR were identical to those of RT-qPCR, except that the probe and ROX reference dye were omitted and replaced with water. The PCR temperature profiles were performed as described in the original papers [35, 36]. PCR products were separated on a 2% agarose gel, stained with the GelGreen DNA dye (Biotium, Hayward, USA), and visualized under blue light excitation. RNA from T-47D cells was included as a positive control for the PGR RT-PCR. The RPL27 gene was used as internal standard to measure the RNA input quantity.

### 2.4. Sample Preparation for Immunohistochemical Analysis

Spheroids were resuspended in 1 mL 4% buffered formaldehyde and stored at 4°C for 48 hours. The fixed spheroids were centrifuged as described above and the pellet was overlaid with 500 *μ*L Histogel® (Richard-Allan Scientific, Microm International, Walldorf, Germany; as specified by the manufacturer). The solidified spheroid pellet was subsequently embedded in Paraplast® (Vogel, Giessen, Germany) by means of an automatic embedding device. Serial sections of 3 *μ*m thickness were cut and either stained with hematoxylin and eosin (H&E) according to Romeis [37] for morphological analyses or mounted on 3-aminopropyltriethoxysilane/glutaraldehyde-coated slides for the different immunohistochemical analyses.

### 2.5. Immunohistochemistry

Endogenous peroxidase activity was blocked by incubation in 0.6% H_2_O_2_ in methanol for 15 min at room temperature. A protein block (1.5% normal goat serum) was used to minimize unspecific binding of the primary antibody. The unlabeled primary antibodies (anti-Ki67, anti-PAIRBP1, anti-PAQR7, and anti-PGRMC1; for sources, pretreatments, and dilutions, see Supplemental Table 2) were detected with the ImmunoVision secondary system (ImmunoVision Technologies, Brisbane, CA, USA) using 3,3′-diaminobenzidine-tetrahydrochloride substrate in Tris-HCl buffer pH 7.4 and 0.03% H_2_O_2_ as chromogen. Finally, slides were washed with distilled water, counterstained with haemalum, dehydrated, and mounted by use of xylene-soluble medium (DPX, Fluka, Buchs, Switzerland). For the double immunofluorescent detection of PGRMC1 and PAIRBP1, Alexa Fluor*™* 488 and Alexa Fluor*™* 568 goat anti-mouse (Molecular Probes, Eugene, OR, USA; dilution 1 : 100) secondary antibodies were used with the UltraVision Quanto Mouse on Mouse HRP Blocking (Thermo Fisher, TL-060-QHDM) in between the two different antibodies for 30 min on room temperature. Nuclear counterstaining was performed with 4′,6-diamidino-2-phenylindole (Molecular Probes/Life Technologies, Vienna, Austria). Negative controls were performed by substituting the primary antibodies with PBS. Sections for the establishment of the immunohistochemical protocols on canine tissue and sections of T-47D tumor cells were kindly provided by the Vetmeduni VetBiobank, VetCore Facility for Research (Vienna, Austria).

Evaluation of the sections was performed using light microscopy (Polyvar, Reichert-Jung, Vienna, Austria) and confocal laser scanning microscopy (Zeiss, LSM 510 Meta, Vienna, Austria).

### 2.6. Scoring System for Proliferative Activity

Proliferative activity was assessed by nuclear anti-Ki67 staining. The nuclei positive for Ki67 protein were counted in three spheroid cross sections per section by using three different sections and four biological replicates per group, resulting in a total number of 36 spheroid cross sections per group. Proliferative activity was determined as the percentage of Ki67-positive cells of total cell number counted per group (scoring index).

### 2.7. Western Blot

Frozen spheroids or PBS-washed fresh cells were lysed using ice-cold lysis buffer (10 mM Tris-HCl pH 7.5, 100 mM NaCl, 1 mM EDTA, 1 mM EGTA, 1% Triton X-100, 10% Glycerol, 0.1% SDS, and 0.5% Na-deoxycholate) supplemented with 1% (v/v) of Protease Inhibitor Cocktail and Phosphatase Inhibitor Cocktail 3 (both from Sigma-Aldrich) using intensive up-and-down pipetting to promote lysis. Samples were then incubated on ice for 30 min, with occasional vortexing. Afterwards, lysates were pushed through 20 G needle several times to shred the DNA followed by centrifugation for 15 min at 4°C and 10 000 ×g. The supernatant fraction was stored at −80°C until further analysis.

Protein extracts (20 *μ*g protein/lane) were separated on 12.5% (PGRMC1) or 10% (PAQR7, PAIRBP1) polyacrylamide minigels for SDS-PAGE electrophoresis under reducing conditions and transferred to PVDF membrane (GE Healthcare, UK). Membranes were blocked using Western Blocking Reagent (Roche Diagnostics, Germany; dilution 1 : 10 in TBST) for two hours at room temperature and probed with the respective primary antibody at 4°C overnight. Afterwards, the membranes were incubated with the respective, species-specific secondary antibodies (peroxidase-linked) for 30 min at room temperature. Proteins were visualized using Amersham Western Blotting Analysis System (GE Healthcare). For negative controls, the membranes were processed in the same way as described above, omitting the respective primary antibody. The primary antibodies for PGRMC1, PAQR7, and PAIRBP1 were the same as indicated in Supplemental Table 2 (respective dilutions of 1 : 1000, 1 : 200, and 1 : 2000). The Amersham ECL-anti-mouse IgG peroxidase-linked species-specific whole antibody from sheep (GE Healthcare, cat. number NA931; dilution 1 : 5000) and Amersham ECL-anti-rabbit IgG peroxidase-linked species-specific whole antibody from donkey (GE Healthcare, cat. number NA934; dilution 1 : 5000) were used as secondary antibodies. All antibodies were diluted in Western Blocking Reagent/TBST (1 : 10).

Postimmunodetection, films, and membranes (poststained with Coomassie R-250) were scanned with an Image Scanner III (GE Healthcare Life Sciences) and quantified by densitometric analysis using Quantity One software (version 4.4.0, Bio-Rad). Coomassie protein staining was used as a loading control and for normalization.

### 2.8. Statistical Analysis

Data are presented as means ± SD. Statistical analysis between comparable groups was performed using unpaired Student's *t*-test [38]. A value of *p* < 0.05 was considered statistically significant.

## 3. Results

### 3.1. Spheroid Formation and Characteristics

**Figure 1:**
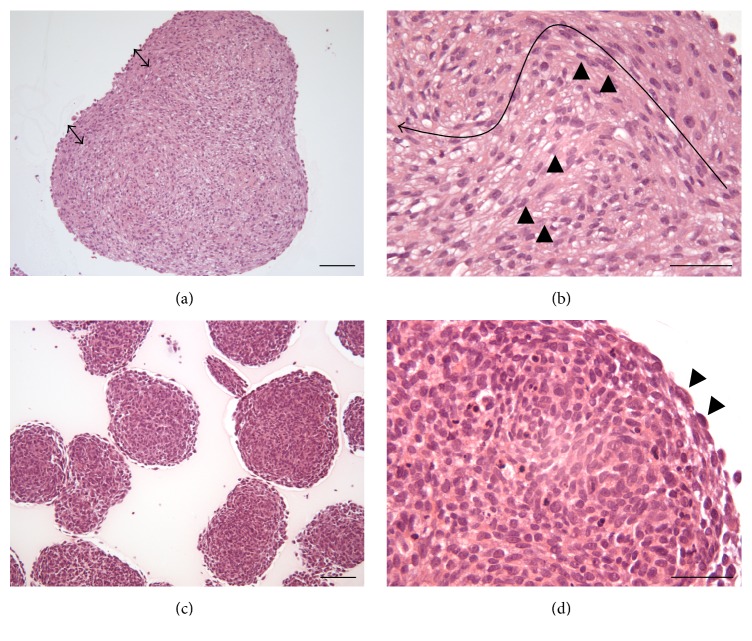
Histological H&E stained sections of U-87 MG ((a), (b)) and LN-229 ((c), (d)) glioma cell spheroids grown for 14 days. (a) The arrows indicate the presence of a capsule-like structure in the periphery of the U-87 MG spheroids. (b) Cells in the periphery were orientated partially in a parallel manner and featured elongated spindle-shaped cells with longish nuclei that occasionally grew into the core of the spheroids (black arrow heads) and formed connective tissue like structures. The elongated arrow indicates the route from the periphery to the core. (c) Spheroids grown from LN-229 cells were less dense than U-87 MG spheroids. (d) The spheroid structure was homogenous and only a thin layer of flat cells was observed circumscribing the spheroid (black arrow heads). Scale bars: (a) and (c) 100 *μ*m, (b) and (d) 50 *μ*m.

The morphological analysis revealed differences in spheroid size, with spheroids grown from U-87 MG cells being 2.6–4.2-fold larger than those from LN-229 cells (Figures 1(a) and 1(c)). The U-87 MG spheroids featured a capsule-like outer region which dispatched lanes of elongated cells with longish nuclei into the core composed of two different cell types: the elongated and round-to-polygonal cells (Figure 1(b)). The outer region of LN-229 spheroids was composed of one layer of flat cells but a differentiation of a capsule-like region from the core tissue was not observed. The nuclei of the LN-229 cells differ partially in size but not in shape (Figure 1(d)).

### 3.2. Effect of Progesterone Treatment on Proliferative Activity

**Figure 2:**
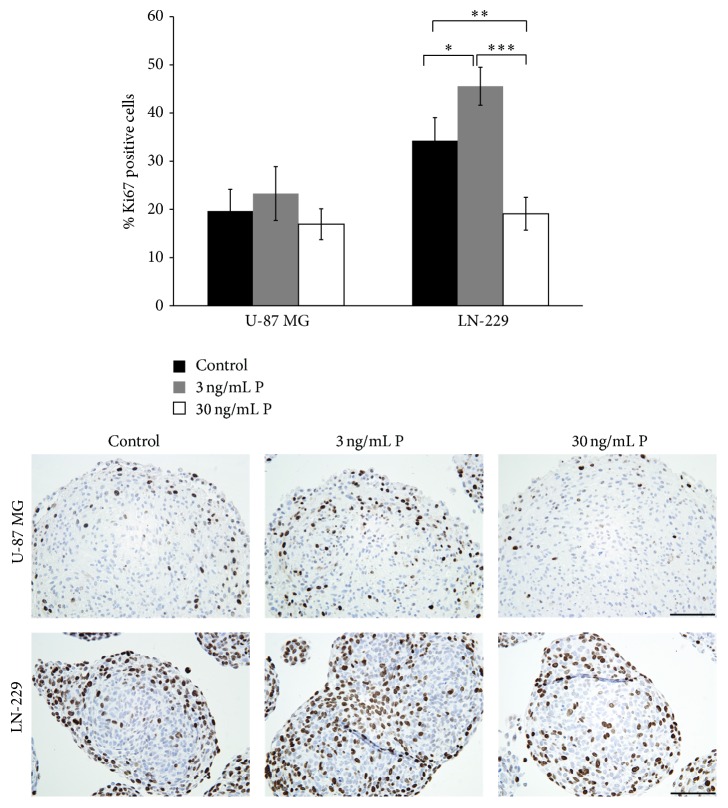
Proliferative activity in U-87 MG and LN-229 glioma spheroids increased by progesterone (P) stimulation using 3 ng/mL P but was reduced by supplementation of 30 ng/mL P. Proliferative activity was assessed by immunohistochemical detection of the Ki67 protein in the two different glioma spheroid types. Scale bars 100 *μ*m. Statistical significance (*p* < 0.05) is indicated by an asterisk (^*∗*^ *p*  0.011; ^*∗∗*^ *p*  0.002; ^*∗∗∗*^ *p*  0.0001).

Proliferative activity of the spheroids of both glioma cell lines increased by stimulation with 3 ng/mL but decreased as a result of 30 ng/mL P supplementation (Figure 2). Even though this trend was observed in both spheroid types, statistically significant differences (*p* < 0.05) were obtained only in LN-229 spheroids. In U-87 MG spheroids' proliferative activity in the control and 30 ng/mL P group was mainly restricted to the outer zone of the spheroid, whereas stimulation with 3 ng/mL increased the number of both peripheral and central cells (Figure 2). In LN-229 spheroids, a distinct concentration of most of the Ki67 positive cells in the spheroid periphery was only observed in the control group, whereas 3 ng/mL P stimulated spheroids featured a high number of Ki67 positive cells in both the periphery and the core (Figure 2).

### 3.3. Effect of Progesterone Treatment on PGRMC1, PAIRBP1, and PAQR7 mRNA Levels

**Figure 3:**
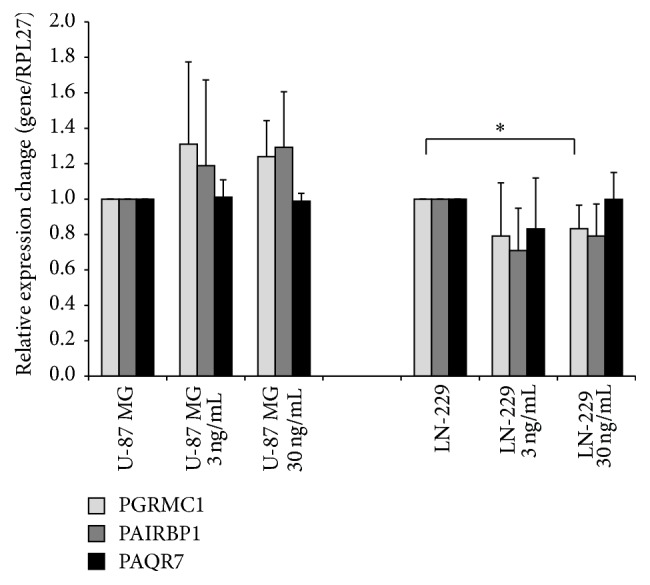
RT-qPCR of PGRMC1, PAIRBP1, and PAQR7 transcripts in U-87 MG and LN-229 glioma spheroids stimulated with 3 ng/mL and 30 ng/mL progesterone, respectively, for 48 hrs. The only significant alteration, decrease in PGRMC1 mRNA level, was observed in LN-229 spheroids treated with 30 ng/mL of P. Statistical significance (*p* < 0.05) is indicated by an asterisk (^*∗*^ *p*  0.046). Data are presented as mean + SD from four independent experiments.

Progesterone treatment of U-87 MG spheroids did not cause any significant changes in PGRMC1, PAIRBP1, and PAQR7 mRNA transcript levels compared to untreated spheroids as shown by the RT-qPCR analysis performed on these samples. However, a positive albeit nonsignificant effect of progesterone on PGRMC1 and PAIRBP1 mRNA levels in U-87 MG spheroids was observed. While the PAQR7 transcript levels were unaffected by progesterone in LN-229 spheroids, a negative trend in PAIRBP1 and PGRMC1 mRNA levels was observed with significant decrease (*p* < 0.05) in PGRMC1 transcript amounts in spheroids treated with 30 ng/mL progesterone (Figure 3).

### 3.4. Effect of Progesterone Treatment on PGRMC1, PAIRBP1, and PAQR7 Protein Levels

**Figure 4:**
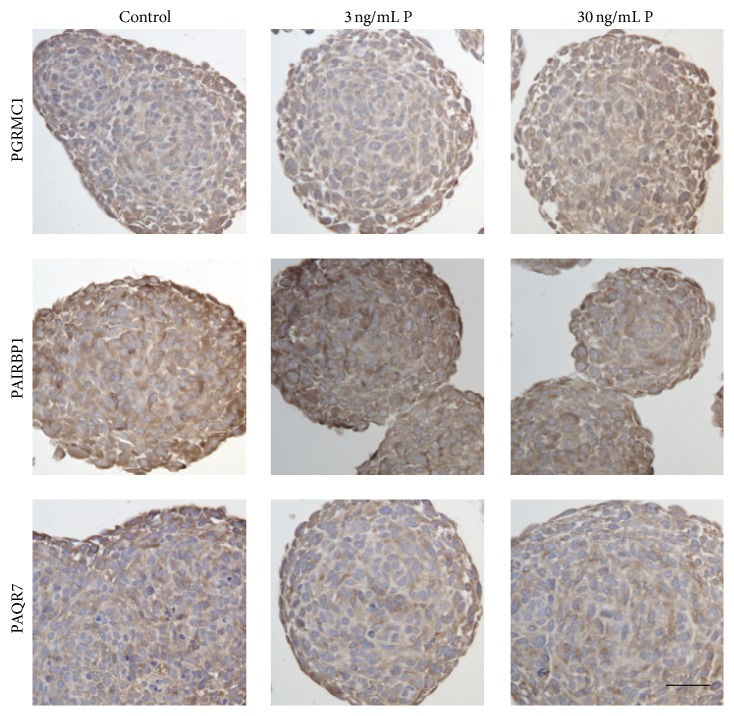
Immunohistochemical detection of PGRMC1, PAIRBP1, and PAQR7 proteins in LN-229 glioma spheroids stimulated with 3 ng/mL and 30 ng/mL progesterone, respectively, for 48 hrs. P supplementation did not induce any alterations in the spheroids compared to the unstimulated control. Scale bar 25 *μ*m.

In the immunohistochemical analysis, LN-229 and U-87 MG spheroids were investigated for the expression of PGRMC1, PAIRBP1, and PAQR7 protein. Human breast cancer cells (MCF-7) served as positive control for establishing the immunohistochemical staining for PGRMC1, PAIRBP1, and PAQR7 (Supplemental Figure 1). In the LN-229 spheroids, neither the stimulation with 3 ng/mL P nor the stimulation with 30 ng/mL P induced any visible alterations in the expression of the three proteins of interest (Figure 4). PGRMC1 and PAIRBP1 were homogenously expressed within the spheroids. PAQR7 protein expression was mainly identified in the cells of the spheroid periphery and in single cells distributed all over the spheroid cross section.

**Figure 5:**
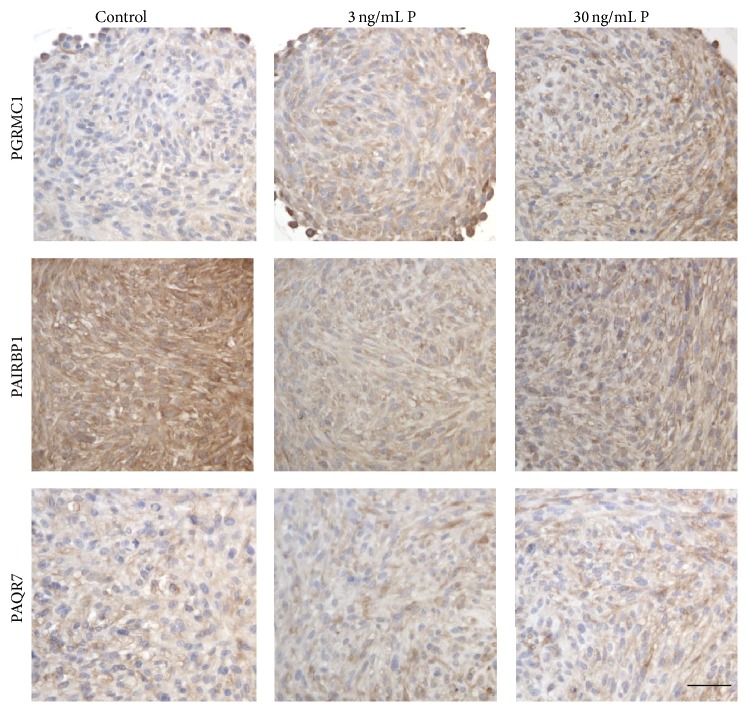
Identification of PGRMC1, PAIRBP1, and PAQR7 protein expression in U-87 MG glioma cell spheroids after 48 hrs progesterone stimulation by means of immunohistochemistry. PGRMC1 protein expression slightly increased through P stimulation, whereas PAIRBP1 expression was decreased. PAQR7 protein expression was unaffected by P supplementation. Scale bar 25 *μ*m.

Stimulation of U-87 MG spheroids with both 3 and 30 ng/mL P resulted in an increase of PGRMC1 protein but in decreased levels of PAIRBP1 compared to the control group. In contrast, PAQR7 was unaffected by P stimulation (Figure 5). PAIRBP1 and PAQR7 protein expression were observed homogenously through the spheroids.

**Figure 6:**
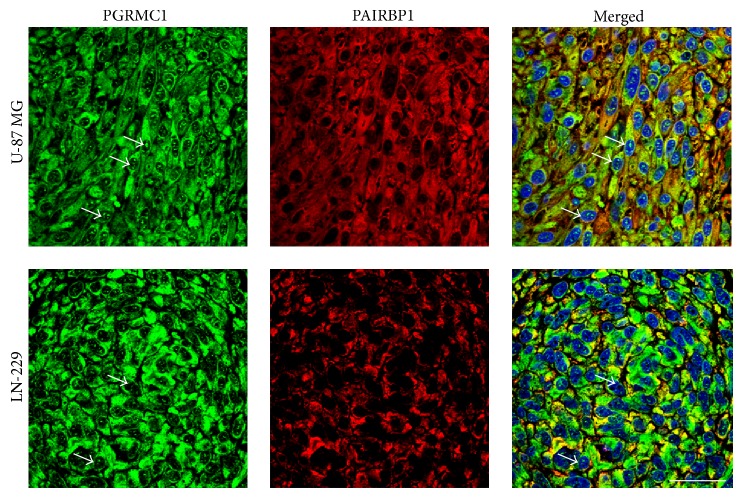
Demonstration of the colocalization of PGRMC1 (green) and PAIRBP1 (red) proteins in U-87 MG and LN-229 glioma cell spheroids by means of double-immunofluorescence. Regions of colocalization are demonstrated by yellow color indicating merged red and green signals. In both cell lines, nuclear PGRMC1 staining (white arrows) was observed. Nuclear counterstaining was performed with 4′,6-diamidino-2-phenylindole (blue). Scale bar 20 *μ*m.

Double immunofluorescent detection of PGRMC1 and PAIRBP1 identified the two proteins to be colocalized in the cells of the spheroids of both glioma cell lines (Figure 6). Stimulation of the spheroids with P in different concentrations did not affect the colocalization pattern of the two proteins (not shown).

**Figure 7:**
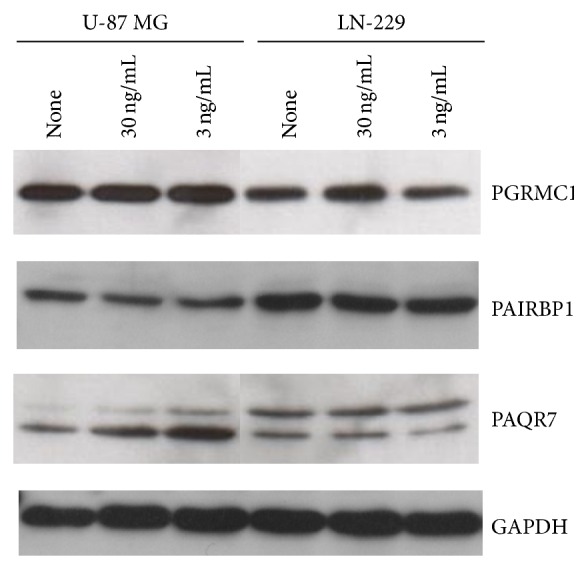
Western blot detection of PGRMC1, PAIRBP1, and PAQR7 protein expression in U-87 MG and LN-229 glioma cell spheroids after 48 hrs progesterone (P) stimulation. Whereas PGRMC1 and PAIRBP1 protein detection revealed the expected band at about 27 kDa and 58 kDa, respectively, two bands migrating at about 48 kDa and 55 kDa were detected using the PAQR7 antibody. A representative Western blot is shown.

In the next step, LN-229 and U-87 MG spheroids were analyzed for the expression of PGRMC1, PAIRBP1, and PAQR7 proteins by Western blot using the same antibodies as applied in the immunohistochemical analysis. Stimulation of U-87 MG as well as LN-229 spheroids with both 3 and 30 ng/mL P did not reveal any changes in PGRMC1 (~27 kDa) and PAIRBP1 (~58 kDa) protein expression (Figure 7). However, detected PGRMC1 levels were higher in U-87 MG spheroids while the PAIRBP1 protein was more abundant in LN-229 spheroids. Interestingly, detection of PAQR7 protein provided two signals at ~48 kDa and ~55 kDa in all spheroids tested. Whereas the ~48 kDa protein was predominant in U-87 MG spheroids, in LN-229 spheroids, the ~55 kDa form displayed a more intense band. In addition, no changes in bands intensities were observed in LN-229 spheroids, irrespective of the progesterone treatment. On the contrary, progesterone stimulation of the U-87 MG spheroids had a positive effect on PAQR7 expression as demonstrated by increasing intensity of both ~48 kDa and ~55 kDa bands as compared to the unstimulated spheroids.

**Figure 8:**
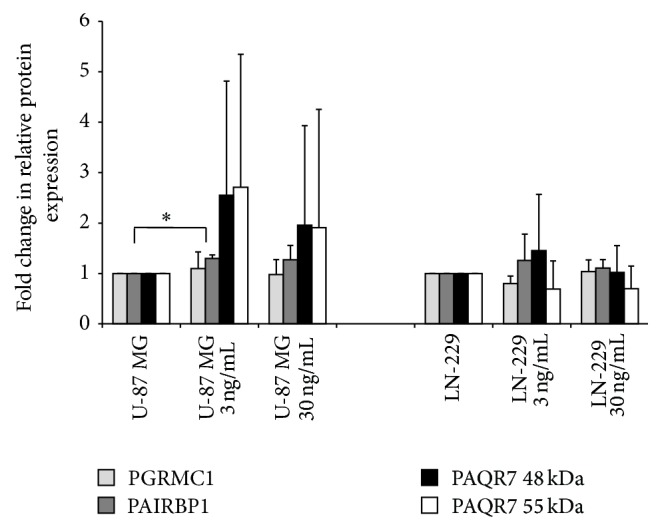
Densitometric analysis of protein expression detected by Western blot. Shown is a fold change in relation to protein amount upon progesterone stimulation. Data are based on protein amount normalized onto the total protein staining in the respective lane. Statistical significance (*p* < 0.05) is indicated by an asterisk (^*∗*^ *p*  0.018). Data are presented as mean + SD from three independent experiments.

Semiquantitative densitometric evaluation of PGRMC1, PAIRBP1, and PAQR7 proteins based on three independent sets of Western blot analysis revealed no statistically significant (*p* < 0.05) changes in respective protein expression in nonstimulated versus progesterone-stimulated LN-229 spheroids. The only statistically significant difference in protein expression was observed for PAIRBP1 expression in nonstimulated versus 3 ng/mL of P stimulated U-87 MG spheroids (Figure 8). A nonsignificant, positive effect was also observed in U-87 MG spheroids treated with 30 ng/mL of progesterone.

### 3.5. Analysis of Nuclear Progesterone Receptor Expression

The expression of the nuclear progesterone receptor was investigated by RT-PCR detecting all known nPGR transcript isoforms A–D [35, 36]. The U-87 MG and LN-229 spheroids, both nonstimulated and progesterone-stimulated, were negative for nPGR transcripts (Supplemental Figures 2(a) and 2(b)). The accuracy of the RT-PCR setup as well as the quality of the isolated RNA of all samples was proven by the presence of nPGR signal in the positive control cells (T-47D) and signal corresponding to RPL27 expression in all samples tested (Supplemental Figures 2(a), 2(b), and 2(c)). Immunohistochemical detection of nPGR was performed on the corresponding samples as RT-PCR and revealed the absence of nPGR protein expression in both glioma cell lines but detected positive signals in the nuclei of T-47D cells (Supplemental Figure 3).

## 4. Discussion

Although the exact mechanism is not fully elucidated, progesterone is known to have dose-dependent antiproliferative and proapoptotic effects in several tumor types, including neuroblastoma and glioblastoma [8, 39]. To that end, proliferative activity of the U-87 MG as well as LN-229 spheroids was evaluated in the presence and absence of progesterone stimulation. As measured by the Ki67 labelling index, proliferation was increased by stimulation with low concentration of progesterone (3 ng/mL) but decreased at high progesterone concentration (30 ng/mL). Although present in spheroids derived from both glioma cell lines, this effect was stronger and statistically relevant only in LN-229 spheroids. Hence, our results further supported the existence of a dual hormetic effect of progesterone on glioma cell proliferation* in vitro* recently described by Atif et al. [40], irrespective of the technique of cell cultivation. The distribution of anti-Ki67 positive cells determined* via* immunohistochemistry was divergent in the two different glioma spheroid types. In the U-87 MG spheroids positive cells were counted in the periphery and an increased number of proliferative active cells were determined in the core of these spheroids after stimulation with 3 ng/mL P. In contrast, in LN-229 spheroids, anti-Ki67 positive cells were mainly located in the periphery of the spheroids. The peripheral concentration of proliferative active cells in spheroids was also reported in other spheroid types constructed from a variety of different cell types [41, 42].

The proposed progesterone action includes the genomic mediated response* via* nuclear progesterone receptors and the nongenomic action, which can be mediated* via* membrane-associated progesterone receptors. Nuclear PGR expression in human clinical astrocytoma samples was demonstrated by Khalid [43], in which 31 out of 33 glioblastomas were positive for nPGR. Moreover, nPGR positive astrocytomas had a higher Ki67 labelling index than nPGR negative tumors. It is noteworthy that, despite discrepant literature data [44], the U-87 MG as well as LN-229 spheroids in our study were found negative for nPGR expression on both the mRNA and protein level as determined by RT-PCR and immunohistochemistry (Supplemental Figures 2 and 3). This discrepancy might result from the different ways of cell culture, because it is well documented that the same cell line cultured in 3D cell culture models differs in gene expression profiles compared to the same cells grown in monolayer cell cultures [42, 45]. Alternatively, it can also be speculated that in different laboratories different types of U-87 MG are cultured. It is well known that cell lines which are commonly used and wide spread sometimes are contaminated with other cell types or change their pheno- and genotype over time and passaging [46]. To ensure the identity of cells used in our study, the STR analysis using Power Plex® 16 system (Promega) was performed by an independent research/service facility (CellBank Graz, Austria). As expected, results of this analysis clearly confirmed identity of U-87 MG and LN-229 cells (Supplemental Figure 4) [47]. Thus, we conclude that the used U-87 MG and LN-229 cells are negative for nPGR. As a consequence of this observation, we further speculate that the progesterone effect on the cellular proliferation was mediated via membrane-associated progesterone receptors. Therefore, we have documented the existence of membrane-associated progesterone receptors PAQR7 and PGRMC1 and of the proposed PGRMC1-downstream component PAIRBP1 in these spheroids in response to progesterone treatment.

In a Western blot analysis, the PGRMC1 protein is predominantly detected as a ~27 kDa band corresponding to a cytoplasmic form, whereas usually less abundant, higher molecular weight bands (~56 kDa and ~75 kDa) correspond to nuclear localization [22, 48]. Immunohistochemical studies located PGRMC1 in the extracellular surface of the plasma membrane, intracellular membranes, cytoplasm, and nucleus [48, 49]. Western blot analysis of spheroid samples revealed, consistent with Peluso's observations, the presence of a ~27 kDa band corresponding to PGRMC1 as the only positive signal detected through the samples [48]. This signal was constant within the spheroid type and the intensity was independent of progesterone treatment. These data are in accordance with the results of immunohistochemical analysis revealing cytoplasmic PGRMC1 as the predominant form detected in the spheroids (Figure 6). However, in addition to cytoplasmic distribution, a number of scattered signals in the nuclei were identified in both U-87 MG and LN-229 spheroids. We can only anticipate that this nuclear PGRMC1 represents a phosphorylated form of PGRMC1, as it has been described in HeLa cells [50].

The PAIRBP1 protein, also known as SERBP1, is differently expressed throughout the human body with low PAIRBP1 expression levels in the brain [51]. Although the exact mechanism of action is not clear, PAIRBP1 has been implicated in tumorigenicity and resistance to anticancer drugs [52, 53]. In ovarian carcinoma, overexpression of PAIRBP1 was associated with higher tumor grading (Grade III versus Grades II and I tumors), while high metastatic potential was linked to PAIRBP1 overexpression in non-small cell lung cancer cells [54, 55]. The PAIRBP1 gene is one out of ten most influential genes for glioblastoma multiforme development [56]. Despite low expression levels in brain tissue observed by Serce, PAIRBP1 upregulation has been found in GBM [51, 56]. Indeed, U-87 MG and LN-229 spheroids were positive for PAIRBP1 expression, at both mRNA and protein level. Similar to PGRMC1, a statistically nonsignificant positive effect of progesterone on PAIRBP1 mRNA expression was observed in U-87 MG spheroids. However, a marginal but rather negative effect was detected in LN-229 spheroids. Surprisingly, Western blot analysis revealed a stronger signal in LN-229 spheroids, with no significant effect of progesterone on PAIRBP1 expression in LN-229 spheroids. However, the semiquantitative densitometric analysis of the respective band (normalized onto the total protein amount loaded per lane) in Western blot revealed a significant increase in PAIRBP1 expression in U-87 MG spheroids treated with low P concentration. Progesterone stimulation did not have an effect on the PAIRBP1 protein localization in both cell lines determined by immunohistochemistry. Thus, no evidence of progesterone-induced cellular stress was observed, as the PAIRBP1 translocates to nuclear-dominant localization upon stress conditions [57].

Recently, PAQR7 expression has been described in human astrocytoma cell lines U-87 and U-251 [58]. In accordance with this observation, PAQR7 expression was also detected in U-87 MG and LN-229 spheroids at mRNA and protein levels. Surprisingly, using polyclonal rabbit anti-PAQR7 antibody from Sigma, PAQR7 was detected as a doublet signal of ~48 and 55 kDa in both U-87 MG and LN-229 spheroids, which was not previously observed by Valadez-Cosmes, using a different PAQR7 antibody [58]. In addition, a positive effect of progesterone on PAQR7 expression was observed in U-87 MG spheroids, while this effect was absent in those derived from LN-229 cells.

## 5. Conclusion

In glioma spheroids grown from U-87 MG and LN-229 cells, membrane-associated progesterone receptors were identified to be involved in the responsiveness of these microtumors* in vitro* to progesterone in a dose-dependent way. As both cell lines were negative for nPGR on protein and mRNA level, we assume that the investigated membrane-associated progesterone receptors are relevant for both the genomic and the nongenomic action of progesterone in the investigated glioma cells. Thus, further investigation of these membrane-associated receptors is necessary to elucidate the function of progesterone action in glioblastoma growth and development and also to reveal their potential for novel therapeutic strategies.

## Supplementary Material

The primers used for the RT-qPCR and RT-PCR analyses have either been described previously, or designed using the PrimerExpress 2.0 software, as specified in the Materials and Methods section (Suppl. Table 1).
In the Suppl. Table 2 sources, pre-treatment of the sections and dilutions of the antibodies used in immunohistochemical analysis are summarized.
In order to establish immunohistochemical staining for PGRMC1, PAIRBP1 and PAQR7, human breast cancer cells (MCF-7) were stained as described in Materials and Methods section using the antibodies listed in Suppl. Table 2. (Suppl. Fig. 1). For PAIRBP1 only cytoplasmic staining was observed whereby PGRMC1 was present in both, the cytoplasm and the nucleus. PAQR7 featured a distinct perinuclear staining pattern. Negative controls were performed by omitting the primary antibody for each protein of interest.
To investigate the nuclear progesterone expression, RT-PCR analysis on RNA samples from non-stimulated as well as progesterone stimulated U-87MG and LN-229 cells was performed. nPGR transcript isoforms A, B, C (Suppl. Fig. 2a) and A, B, D (Suppl. Fig. 2b) were investigated. Positive signal at 121bp (isoforms A, B, C) or 148bp (isoforms A, B, D) was only detected in T-47D cells used as a positive control. The RNA integrity was confirmed by amplification of 121bp signal corresponding to the RPL gene in all samples tested (Suppl. Fig. 2c).
To further analyze the nPGR expression also on the protein level, U-87 MG and LN-229 spheroids (both non-stimulated as well as progesterone stimulated) were analyzed by means of immunochemistry. Consistent with the RT-PCR data, spheroid samples were negative for nPGR expression, whereas T-47D cells were positive (Suppl. Fig. 3a,b).
Substitution of cell lines used for experimental work is often responsible for discrepancies observed in results published by different laboratories. To ensure the identity of cells used in our study, the STR analysis was performed and confirmed match with U-87 MG and LN-229 cells (Suppl. Fig. 4).

## Abbreviations

bp: Base pair
DMEM: Dulbecco's modified Eagle's medium
GAPDH: Glyceraldehyde-3-phosphate dehydrogenase
GBM: Glioblastoma multiforme
H&E: Hematoxylin and eosin stain
kDa: Kilodalton
MAPR: Membrane-associated progesterone receptors
nPGR: Nuclear progesterone receptors
P: Progesterone
PAIRBP1: Plasminogen activator inhibitor 1 RNA-binding protein
PAQR7: Progestin and adipoQ receptor 7
PCR: Polymerase chain reaction
PGRMC1: Progesterone receptor membrane component 1
RT-PCR: Reverse transcription PCR
SERBP1: SERPINE1 mRNA-binding protein.
