# Supplementary material for: Expression of Progesterone Receptor Membrane Component 1 (PGRMC1), Progestin and AdipoQ Receptor 7 (PAQPR7), and Plasminogen Activator Inhibitor 1 RNA-Binding Protein (PAIRBP1) in Glioma Spheroids In Vitro
Source: Biomed Res Int. 2016 Jun 1;2016:8065830. doi: 10.1155/2016/8065830 (PMC4908248; doi:10.1155/2016/8065830)
Supplement: Supplementary file 1 — The primers used for the RT-qPCR and RT-PCR analyses have either been described previously, or designed using the PrimerExpress 2.0 software, as specified in the Materials and Methods section (Suppl. Table 1). In the Suppl. Table 2 sources, pre-treatment of the sections and dilutions of the antibodies used in immunohistochemical analysis are summarized. In order to establish immunohistochemical staining for PGRMC1, PAIRBP1 and PAQR7, human breast cancer cells (MCF-7) were stained as described in Materials and Methods section using the antibodies listed in Suppl. Table 2. (Suppl. Fig. 1). For PAIRBP1 only cytoplasmic staining was observed whereby PGRMC1 was present in both, the cytoplasm and the nucleus. PAQR7 featured a distinct perinuclear staining pattern. Negative controls were performed by omitting the primary antibody for each protein of interest. To investigate the nuclear progesterone expression, RT-PCR analysis on RNA samples from non-stimulated as well as progesterone stimulated U-87MG and LN-229 cells was performed. nPGR transcript isoforms A, B, C (Suppl. Fig. 2a) and A, B, D (Suppl. Fig. 2b) were investigated. Positive signal at 121bp (isoforms A, B, C) or 148bp (isoforms A, B, D) was only detected in T-47D cells used as a positive control. The RNA integrity was confirmed by amplification of 121bp signal corresponding to the RPL gene in all samples tested (Suppl. Fig. 2c). To further analyze the nPGR expression also on the protein level, U-87 MG and LN-229 spheroids (both non-stimulated as well as progesterone stimulated) were analyzed by means of immunochemistry. Consistent with the RT-PCR data, spheroid samples were negative for nPGR expression, whereas T-47D cells were positive (Suppl. Fig. 3a,b). Substitution of cell lines used for experimental work is often responsible for discrepancies observed in results published by different laboratories. To ensure the identity of cells used in our study, the STR analysis was performed and confirmed match with U [file 8065830.f1.pdf]

Supplementary Figure 1. Human breast cancer cells (MCF-7) served as positive control for establishing the immunohistochemical staining for PGRMC1, PAIRBP1 and PAQR7. For PAIRBP1 only cytoplasmic staining was observed whereby PGRMC1 was present in both, the cytoplasm and the nucleus. PAQR7 featured a distinct perinuclear staining pattern. Negative controls were performed by omitting the primary antibody for each protein of interest. Representative negative controls of MCF-7 sections without any signals are demonstrated for the secondary rabbit (NC rabbit, PGRMC1) and mouse system (NC mouse, PAIRBP1), respectively. Scale bars for all figures 25µm.

Supplementary Figure 2. nPGR isoforms A, B, C (**a**), nPGR isoforms A, B, D (**b**) and RPL 27 (**c**) detection in glioma spheroids using RT-PCR. One microgram of total RNA was reverse-transcribed into cDNA utilizing the High Capacity Reverse Transcription Kit (+RT), whereas samples in which no RT enzyme was added (-RT) were included for all RNAs to monitor for the amplification of residual DNA. Human breast tumor cell line T-47D was taken as a positive control for nPGR expression. NC – negative control without template DNA. Expected PCR products of 121bp (a, c) and 148bp (b) were separated on a 2% agarose gel. Lane 1,17 - DNA marker; lane 2,10 – T-47D; lane 3,11 – U-87 MG; 4,12 – U-87 MG 30 ng/mL progesterone; 5,13 – U-87 MG 3 ng/mL progesterone; 6,14 – LN-229; 7,15 – LN-229 30 ng/mL progesterone; 8,16 – LN-229 3 ng/ml progesterone; 9 - NC.

Supplementary Figure 3. Human glioma spheroids derived from U-87 MG and LN-229 cells were negative for nPGR detection by immunochemistry, irrespective of the progesterone stimulation. Human breast cancer cells (T-47D) served as positive control for nPGR protein expression (left) and the negative control (primary antibody omitted, right) was without staining signals. Scale bars for spheroids 100 µm, T-47D cells 25µm.

Supplementary Figure 4. The STR analysis confirming identity of U-87 MG and LN-229 cell lines. The ATCC has originally stated, that the U-87MG cell line (ATCC# HTB-14) is of male origin (http://www.lgcstandards-atcc.org/products/all/HTB-14.aspx?geo_country=at#generalinformation; acquired 07.01.2016). The gender discrepancy observed in U-87 MG cell line is not unusual. It is possible that the cell line was misidentified in the depositor´s original publication or the Y chromosome got lost during the extensive cultivation process. The cells were obtained in passage 28 from the cell bank (Cell Line Service) and used in passages 35-40.

Supplementary Figure 1

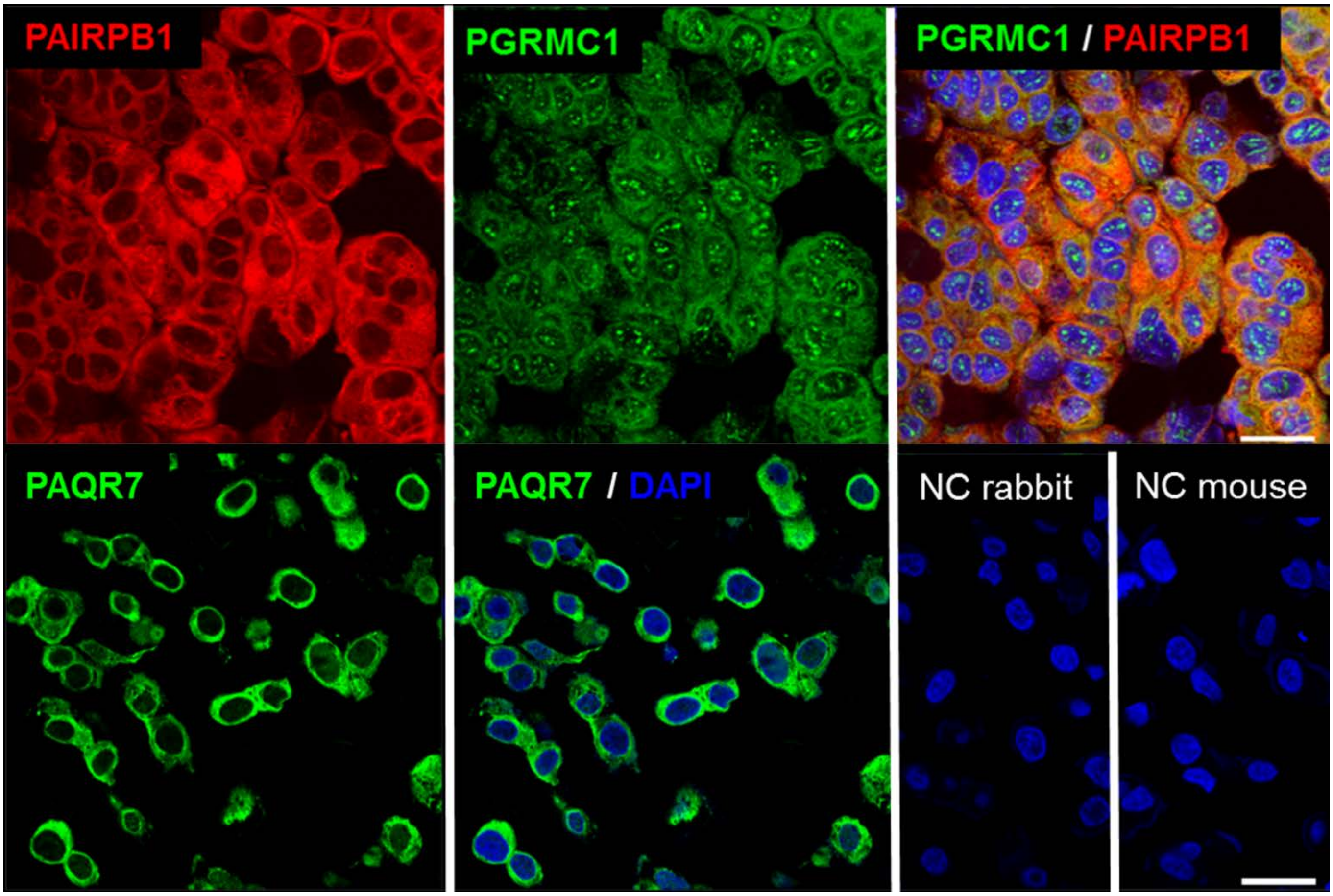

Supplementary Figure 2

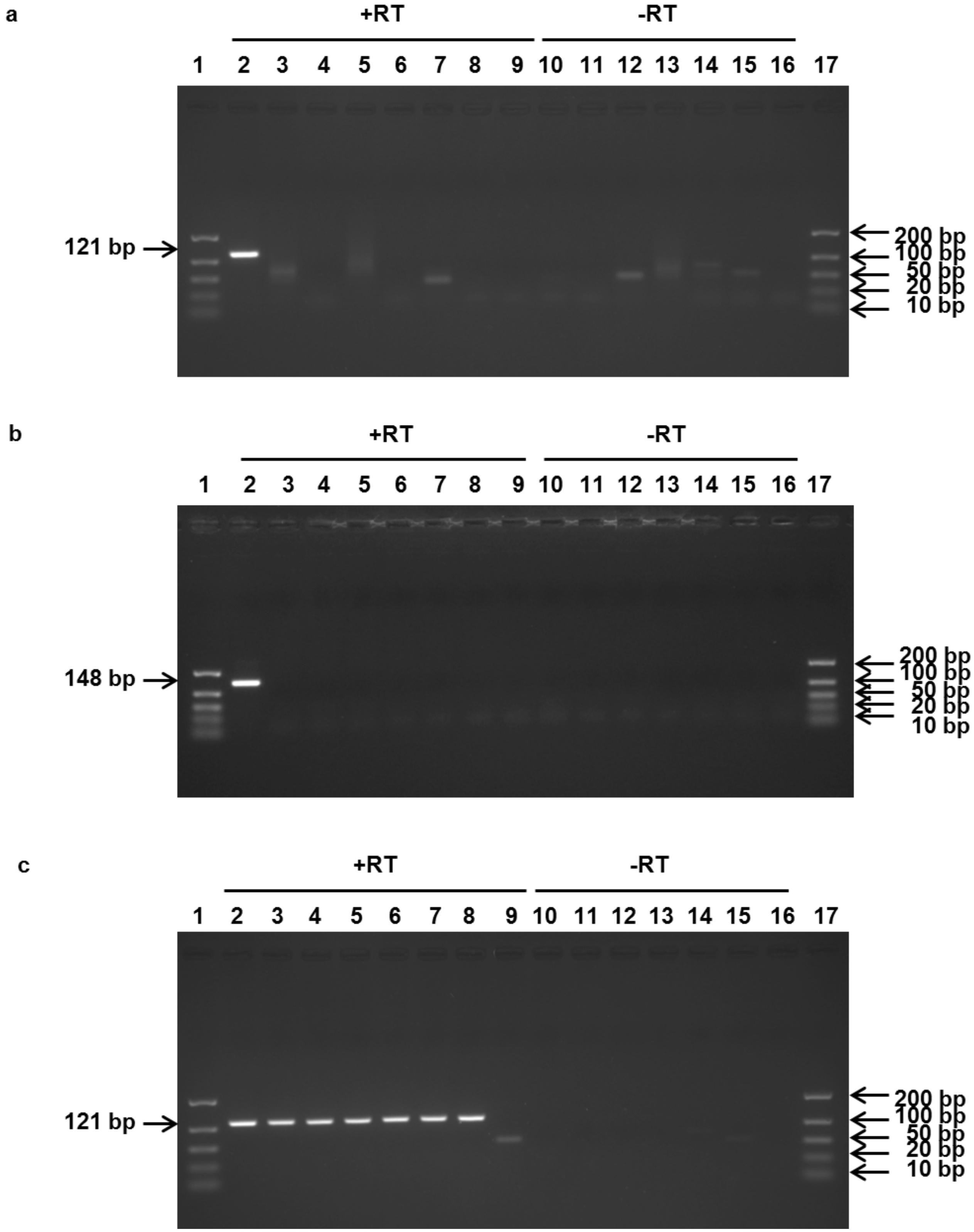

Supplementary Figure 3

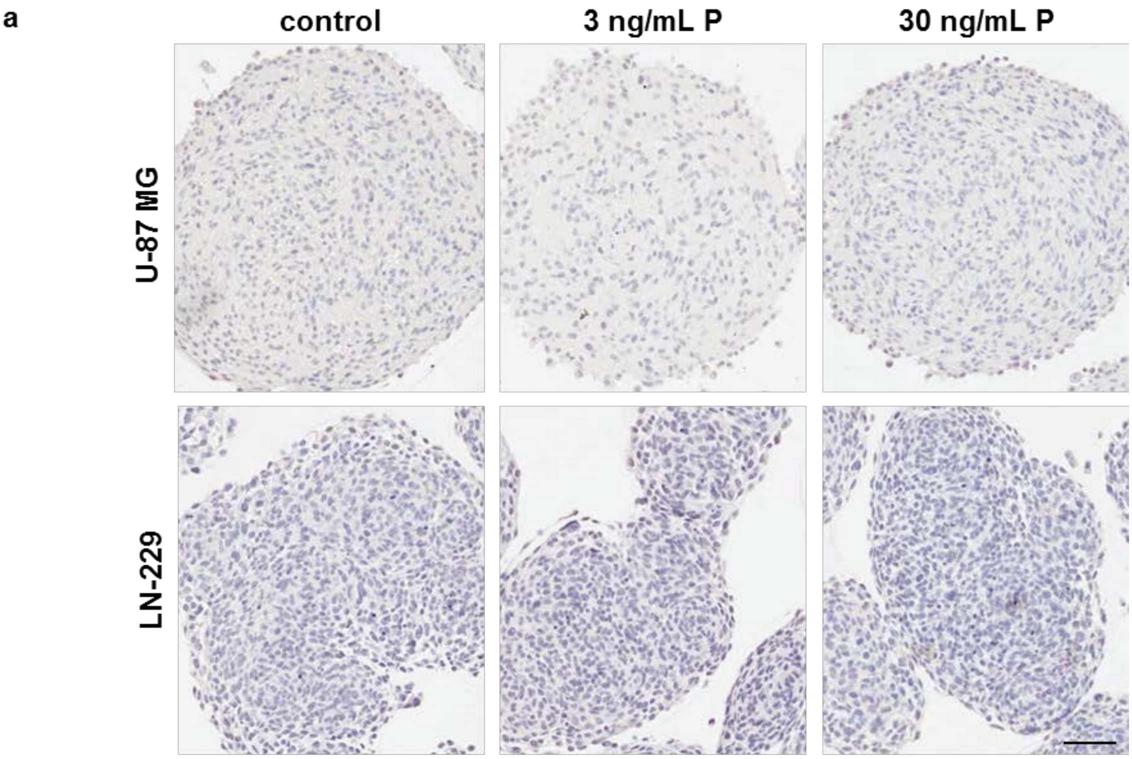

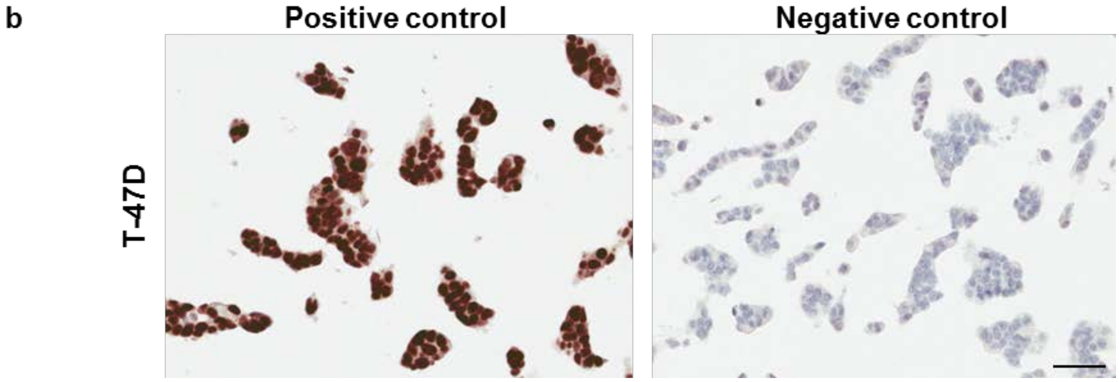

Supplementary Figure 4

Cell Culture Facility

STR-Results for: U87mg	Date: 10.12.2015

cell culture facility

STR-Locus	your cell line	ATCC
D3S1358	16,17	
TH01	9.3	9.3
D21S11	28, 32.2	
D18S51	13,14	
Penta E	7,14	
D5S818	11,12	11,12
D13S317	8,11	8,11
D7S820	8,9	8,9
D16S539	12	12
CSF1PO	10,11	10,11
Penta D	9,14	
Amelogenin	X	X
vWA	15,17	15,17
D8S1179	10,11	
TPOX	8	8
FGA	18,24	

Kit: Promega, PowerPlex 16HS System (Cat.No. DC2101)

Cell Culture Facility

STR-Results for:	Date:
**LN-229**	10.12.2015

STR-Locus	your cell line	ATCC
D3S1358	16,17	
TH01	9.3	9.3
D21S11	29,30	
D18S51	13	
Penta E	7,16	
D5S818	11,12	11,12
D13S317	10,11	10,11
D7S820	8,11	8,11
D16S539	12	12
CSF1PO	12	12
Penta D	10,11	
Amelogenin	X	X
vWA	16,19	16,19
D8S1179	13,14	
TPOX	8	8
FGA	23	

Kit: Promega, PowerPlex 16HS System (Cat.No. DC2101)

Supplementary Table 1. Primers used for RT-qPCR and RT-CR.

Accession number*	Gene symbol	Gene name	Oligo	Sequence (5' – 3')	Amplicon size (bp)	PCR efficiency (%)	Reference
NM_178422.5, XM_011540861.1, XM_005245746.2, XM_011540862.1, XM_005245745.2	PAQR7	Progestin and adipoQ receptor family member VII	Forward<br>Reverse<br>Probe	CGCTCTTCTGGAAGCCGTACATCTATG<br>CAGCAGGTGGGTCCAGACATTCAC<br>FAM-CGCTGCATCAGACCTGGCGCTTCTATTT-BHQ1	122	0.914	[30]
NM_006667.4, NM_001282621.1	PGRMC1	Progesterone receptor membrane component 1	Forward<br>Reverse<br>Probe	CTGCATGATTTCTGTTTTATCTACCTCTA<br>TGTTACTGGACAGCGCTTAATCC<br>FAM-AGCAAATCTGCAGTGTTCCAAAGACTTTGG-BHQ1	86	0.914	-
NM_015640.3, NM_001018067.1, NM_001018068.1, NM_001018069.1	SERBP1	SERPINE1 mRNA binding protein 1	Forward<br>Reverse<br>Probe	GAGTGAAGAGGCTCATGCTGAA<br>CCAGCTGAGACGTTATATCATTTGC<br>FAM-ATTCGGTTATGGACCATCATTTCCGGA-BHQ1	80	0.945	-
NM_000988.3	RPL27	Ribosomal protein L27	Forward<br>Reverse<br>Probe	TCGCCAAGAGATCAAAGATAA<br>CTGAAGACATCCTTATTGACG<br>FAM-ACCTAATGCCCACAAGGTACTCTGT-BHQ1	121	0.932	[31]
NM_000926.4, NM_001202474.3, NM_001271161.2	PGR	Progesterone receptor (isoforms A, B, C)	Forward<br>Reverse	CGCGCTCTACCCTGCACTC<br>TGAATCCGGCCTCAGGTAGTT	121	N/A	[35]
NM_000926.4, NM_001202474.3, NM_001271162.1	PGR	Progesterone receptor (isoforms A, B, D)	Forward<br>Reverse	AGCATGTCGCCTTAGAAAGTGC<br>TAGGGCTTGGCTTTCATTTG	148	N/A	[36]

FAM = 6-carboxyfluorescein, BHQ1 = Black hole quencher 1

*National Center for Biotechnology Information (NCBI), Entrez Gene (http://www.ncbi.nlm.nih.gov/sites/entrez?db=gene)

N/A = not available

Supplementary Table 2. Sources, pre-treatment of the sections and dilutions of the antibodies used in immunohistochemical analysis.

Antibody	Host and clone	Source	Pre-treatment	Dilution
anti-Ki67	mouse monoclonal 7B11	Invitrogen, CA, USA	boil in citrate buffer pH 6.0 4x5min	1:100
anti-nPGR	rabbit monoclonal Y85	Cell Marque, Rocklin, USA	boil in Tris-EDTA (Dako) pH 9.0 3x5min	1:50
anti-PAIRBP1	mouse monoclonal 4G2	Abcam, Cambridge, UK	boil in Tris-EDTA (Dako) pH 9.0 3x5min	1:1000
anti-PGRMC1	rabbit polyclonal	Prestige Antibodies, Sigma-Aldrich, St. Louis MO, USA	boil in citrate buffer pH6.0 3x5min	1:500
anti-PAQR7	rabbit polyclonal	Prestige Antibodies, Sigma-Aldrich, St. Louis MO, USA	boil in citrate buffer pH6.0 3x5min	1:100
